# Sequential infections with rhinovirus and influenza modulate the replicative capacity of SARS-CoV-2 in the upper respiratory tract

**DOI:** 10.1080/22221751.2021.2021806

**Published:** 2022-01-27

**Authors:** Manel Essaidi-Laziosi, Catia Alvarez, Olha Puhach, Pascale Sattonnet-Roche, Giulia Torriani, Caroline Tapparel, Laurent Kaiser, Isabella Eckerle

**Affiliations:** a Department of Microbiology and Molecular Medicine, Faculty of Medicine, University of Geneva, Geneva, Switzerland; b Geneva Centre for Emerging Viral Diseases, Geneva University Hospitals, Geneva, Switzerland; c Laboratory of Virology, Division of Infectious Diseases and Division of Laboratory Medicine, University Hospitals of Geneva, University of Geneva, Geneva, Switzerland; d Division of Infectious Diseases, Geneva University Hospitals, Geneva, Switzerland

**Keywords:** SARS-CoV-2, covid-19, rhinovirus, viral co-infections, influenza virus, interferon, variants of concern, Alpha variant

## Abstract

Although frequently reported since the beginning of the pandemic, questions remain regarding the impact of Severe Acute Respiratory Syndrome Coronavirus 2 (SARS-CoV-2) interaction with circulating respiratory viruses in coinfected patients. We here investigated dual infections involving early-pandemic SARS-CoV-2 and the Alpha variant and three of the most prevalent respiratory viruses, rhinovirus (RV) and Influenza A and B viruses (IAV and IBV), in reconstituted respiratory airway epithelial cells cultured at air–liquid interface. We found that SARS-CoV-2 replication was impaired by primary, but not secondary, rhino- and influenza virus infection. In contrast, SARS-CoV-2 had no effect on the replication of these seasonal respiratory viruses. Inhibition of SARS-CoV-2 correlated better with immune response triggered by RV, IAV and IBV than the virus entry. Using neutralizing antibody against type I and III interferons, SARS-CoV-2 blockade in dual infections could be partly prevented. Altogether, these data suggested that SARS-CoV-2 interaction with seasonal respiratory viruses would be modulated by interferon induction and could impact SARS-CoV-2 epidemiology when circulation of other respiratory viruses is restored.

## Introduction

The emergence of the Severe Acute Respiratory Syndrome Coronavirus 2 (SARS-CoV-2), the causative agent of coronavirus disease 2019 (COVID-19) has led to a pandemic and an unprecedented health crisis. Even out of the pandemic context, respiratory viral infections are among the most frequent infectious diseases world-wide and constitute a global public health concern especially in vulnerable persons [1]. Respiratory viruses belong to a large range of taxonomically different groups which are able to infect the human respiratory tract epithelium (reviewed in [2]). In particular, rhinoviruses (RVs), are the most prevalent respiratory viruses and are the leading cause of mild common cold disease. Seasonal influenza A (IAV) and B (IBV) viruses can lead to more severe or even fatal illnesses, with clinical presentation and at-risk populations comparable to COVID-19.

Several studies have recently reported the co-detection of SARS-CoV-2 with additional respiratory viruses in co-infected children and adults [3–5]. However, conflicting results were found in terms of clinical relevance [6,7]. Questions remain regarding the impact of co-infections on virus replication and disease severity and the mechanisms involved. Most of conclusions drawn from clinical data from the first pandemic wave in early 2020 might be biased for two reasons. First, the majority of these data have been recorded from hospitalized patients with severe diseases, while co-infected patients with mild/without respiratory symptoms would be under-represented in these studies. Second, due to the hygiene measures implemented in many parts of the world, the overall prevalence of respiratory viruses, especially enveloped viruses in temperate areas, dropped dramatically during the COVID-19 pandemic [8,9]. In both the Northern and Southern hemisphere, the nearly complete absence of seasonal influenza in 2020/2021 has been observed by the national surveillance programs [10–12]. Meanwhile, new SARS-CoV-2 variants of concern have emerged, like the Alpha variant (B.1.1.7) which quickly outcompeted earlier strains in early 2021, then followed by Delta in mid 2021 [13,14]. Considering the expansion of vaccination rates in vulnerable and non-vulnerable populations, and the pressing need to loosen current restrictions, a resurge in respiratory viruses is observed in the current winter season [10–12]. This leads to the ongoing co-circulation of SARS-CoV-2, including its variants, and other respiratory viruses. Also, co-infections might be more frequently observed in the future, especially with SARS-CoV-2 becoming firmly established in the human population [11,15], and would play an important role in disease course in patients and SARS-CoV-2 epidemiology. Thus, there is an urgent necessity to understand how SARS-CoV-2 interacts with other respiratory viruses and the consequence of this interaction.

Morphologically and functionally close to the airway epithelium, reconstituted primary airway epithelial cells (HAE) cultured in air–liquid interface (ALI) are a suitable surrogate model to recapitulate the *in vivo* situation and study viral infections of the respiratory tract and the mechanisms implicated in respiratory virus-virus and host-virus interactions during single and co-infections [16–21]. To investigate the influence of respiratory co-infections on virus replication, here we have assessed co-infections of the first wave SARS-CoV-2 (already harboring the spike mutation at position D614G) and the SARS-CoV-2 Alpha variant of concern (VOC) with the pre-pandemic most prevalent respiratory viruses, RV-A, IAV and IBV, in *in vitro* differentiated airway epithelial tissues. We found that SARS-CoV-2 replication is impaired by a primary, but not secondary, infection with seasonal respiratory viruses. Virus-virus interaction would be modulated by interferon induction and additional host cells response mechanisms. This work has allowed a better understating of molecular and cellular pathways implicated during airway epithelium coinfections, which would impact SARS-CoV-2 circulation in a seasonal manner after the pandemic once circulation of other respiratory viruses will be restored.

## Materials and methods

### Viruses

The viruses used in this study are summarized in table S1. All these viruses were isolated and produced directly from clinical samples (nasopharyngeal swabs) in HAE as previously described [16,17] to avoid any adaptation in standard cells.

### Human in vitro differentiated airway epithelia

All single and dual infections were performed in 3-dimentional tissues called “MucilAir™” purchased from Epithelix SARL [www.epithelix.com]. According to the manufacturer, *in vitro* reconstitution was performed using a mixture of dedifferentiated epithelial cells obtained from nasal polyps of 14 patients. Cells were cultured in transwells at 37°C and 5% CO_2_ for about 4–5 weeks at ALI conditions (more details in [16]). In this culture, MucilAir^TM^ medium (Epithelix) was supplemented (700 µL) from the basal compartment and changed twice a week, while the apical was in contact with air. Once differentiated, the airway epithelial tissue is composed of around 400’000 pseudostratified cells, of which approximately 200’000 are accessible from the apical surface. These tissues are morphologically and functionally close to the airway epithelium in patients. The muco-ciliary clearance is insured by goblet (producing the mucus) and ciliated cells. It is stable during months when cultivated in ALI system at 37°C under a 5% of CO_2_ atmosphere.

### Single and dual infection assays

Infections of nasal airway epithelial tissues cultured in air–liquid interface by respiratory viruses were performed as previously described [16,17]. The multiplicity of infection (MOI) was selected in the range where the virus has the optimal kinetics in single infection for each virus (Table S1). Higher MOI was used for SARS-CoV-2 compared to RV and influenza viruses. Briefly, after 3 h of apical virus inoculation, HAE were washed three times with PBS (Phosphate Buffered Saline, Sigma) and incubated at 33°C and 5% CO_2_. For each time point, 200 µL of MucilAir^TM^ medium were daily added apically and collected after 20 min of incubation at 33°C and 5% CO_2_. For dual infections, similar protocol was used for the second infection 1 or 2 days after the first one. In order to study the involvement of interferon (IFN), similar dual assays were repeated where a mixture of anti-type I neutralizing antibodies diluted 1/50 (39000-1, PBL Assay Science^TM^, Piscataway, NJ, USA) and 10μg/mL of anti-IFN-λ1 (MAB15981-100, RandD, Minneapolis, MN, USA), were added in the basolateral medium as previously described [17,18].

### Viral RNA quantification

Viral load was determined from RNA, extracted with NucliSens easyMAG (BioMérieux), by quantitative real time PCR (RT-qPCR) using SuperScript™ III Platinum™ One-Step qRT-PCR Kit (Invitrogen) in CFX96 Thermal Cycler (BIORAD). Real time RT-qPCR was performed using specific sets of primers and probes as previously described [16,22]. Data were analyzed using Bio-Rad CFX maestro software (BIORAD).

### Host gene induction

Gene inductions of IFN-α and IFN-β, IFN-λ, ISG15 and angiotensin-converting enzyme 2 (ACE-2) were determined by semi-quantitative real-time PCR on total intracellular RNA extracted from tissue lysates obtained using Easymag lysis buffer (BioMérieux 280134). mRNA was amplified using specific gene expression assay kits purchased from Life Technology (4331182). The induction of these genes in infected tissues was represented in fold change relative to non-infected tissue and normalized to a housekeeping gene, RNAseP (Life Technology 4331182).

### Immunofluorescence

**Figure 1.**
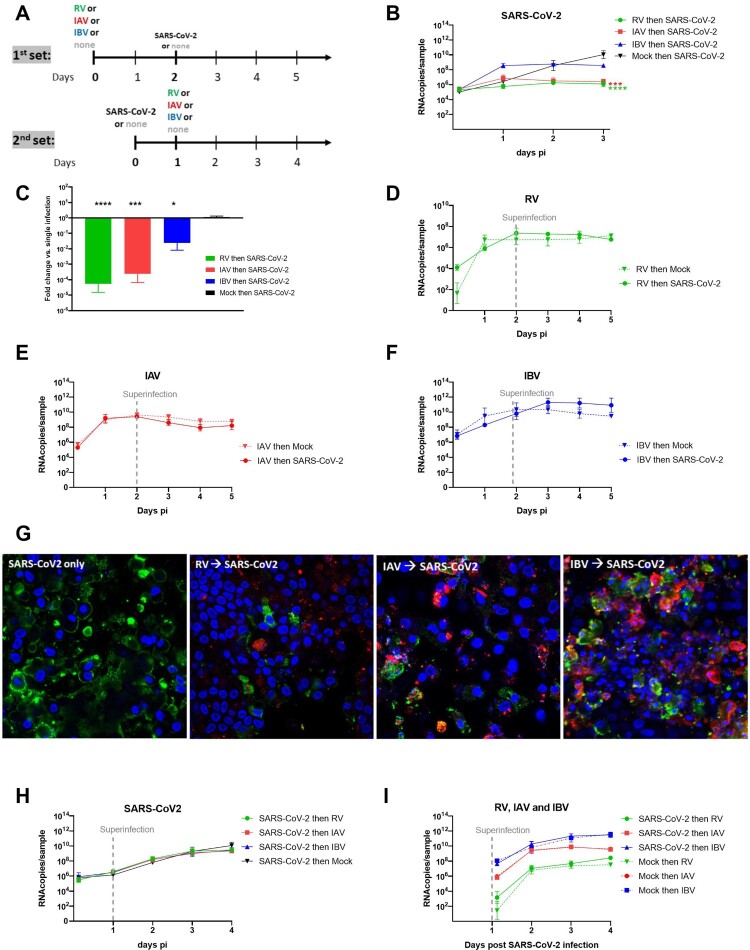
Coinfection assays. A. Two sets of coinfections assays were performed by varying the sequence of infections. In the first set (upper panel): RV/IAV/IBV then, 2days after, SARS-CoV-2. In the second set (lower panel) SARS-CoV-2 then, 1day after, RV/IAV/IBV. Single infections were tested in parallel as control. Viral replication was quantified from apically released virus by RT-qPCR. B. SARS-CoV-2 replication in the first condition (N = 6). C. Fold change in apically released virus at 3days post SARS-CoV-2 infection in dual relative to single infection is represented (from the same experiments as in B) (N = 6). D., E. and F. RV, IAV and IBV replication, respectively, in single Infection (dotted lines) and in the presence of SARS-CoV-2 secondary infection (Solid lines) (N = 3-4). G. Immuno-fluorescence in coinfected tissues at day5. Coinfection assays, as schemed in the upper panel of figure1A were performed. Green: SARS-CoV-2. Red: RV or IAV or IBV. Blue: nuclei. Yellow: coinfected cells. Of note, we here selected images showing coinfected cells even if this event is rarely detected. H. SARS-CoV-2 replication in single infection versus tissues post-infected by RV, IAV and IBV (N = 4). I. RV, IAV and IBV replication in single Infection (dotted lines) and in the presence of SARS-CoV-2 pre-infection (Solid lines). Data are expressed as mean and SEM (N = 4). In comparison to single infection, statistical significance was calculated with t-tests on the area under the curve from kinetics of virus replication (B, D, E, F, G and H) and using one-way ANOVA for fold change (C). *P < 0.05, ***P < 0.001 and ****P < 0.0001.

At the end of the co-infection (at day 5 corresponding to 3days post SARS-CoV-2 superinfection), as schemed in the upper panel of Figure 1A were performed as previously described [16]. Briefly, co-infected tissues were fixed for 30 min in 4% paraformaldehyde (PAF) at room temperature (RT), washed 3 times with PBS, permeabilized with Perm/wash buffer (BD 554723) and co-stained to detect infected cells using antibodies against VP3 RV (Thermofisher G47A, MA5-18249), IAV (Merk 5001), IBV (Merk 5002), NP SARS-CoV-2 (Rockland 200-401-50) and β-tubulin IV (abcam / ab179504). Nuclei were stained with 4’, 6-diamino-2-phenylindole (DAPI). Images were acquired using Zeiss LSM 700 Meta confocal microscope with a 63.6/1.4 objective.

### SARS-CoV-2 titration by focus-forming assay

Vero E6 cells (40’000) seeded in monolayer were inoculated at 37°C and 5% CO_2_ with serially diluted supernatant collected from single and dual infections. One hour later, the inoculum was replaced by prewarmed DMEM (10%FBS, 2 mM L-glutamine, 1%penicillin–streptomycin all from Gibco) mixed (1:1) with 2.4% Avicel. After 24H of incubation at 37°C and 5% CO_2_, cells were fixed using 6%PAF at least 1 h at RT, permeabilized with 0.1%Triton X-100 and blocked with 1%Bovin serum albumin (Sigma). Cells were then incubated with a monoclonal anti-SARS-CoV N antibody (JS02 produced by Geneva Antibody facility at the Faculty of Medicine of Geneva) for 1 h at RT and then with peroxidase-conjugated secondary antibody (Jackson ImmunoResearch, 109-036-09) for 30 min at RT. Foci, visualized using True Blue HRP substrate (Avantor) and imaged on an ELISPOT reader (CTL) were counted to determine the number of focus forming units per mL (FFU/mL) for each sample.

## Results

### SARS-CoV-2 replication is inhibited by a concurrent pre-existing infection with RV-A, IAV and IBV but not vice versa

In order to study the effect of respiratory viruses on SARS-CoV-2 replication in multiple versus single infections, sequential infections were performed as previously described [17,18]. In a first set of co-infection assays (Figure 1A, upper panel), RV, IAV and IBV pre-infected tissues were inoculated with SARS-CoV-2 48 h after the primary (first) infection. While SARS-CoV-2 replication in the single infection experiment reached its peak viral load at 10.4 log10 viral RNA copies (RNAc) at 3days post-infection (dpi), SARS-CoV-2 replication was almost completely abolished in RV and IAV pre-infected tissues (on average less than 1 log10 fold change 72hpi/3hpi for RV and IAV) (Figure 1B). In contrast, in IBV pre-infected tissues, SARS-CoV-2 replication was observed but to a lower extent compared to in IAV and RV pre-infected tissues (log10 fold decrease relative to single infection 1.6 versus 3.62 and 4.25, respectively) (Figures 1B and C). In all these sequential infections, interference with SARS-CoV-2 replication started at day 2. Only in IBV pre-infected tissues, was an increase of SARS-CoV-2 replication (6.42 RNAc/sample in single infection versus 8.56 RNAc/sample in IBV-infected tissues) observed already at 1 dpi. In contrast, no effect of SARS-CoV-2 secondary co-infection on the replication of the primary co-infecting virus was seen, neither for RV (7.1 log10 RNAc/sample), nor IAV (8.7 log10 RNAc/sample), nor IBV (10.9 log10 RNAc/sample) (Figures 1D, E and F). Impaired replication of SARS-CoV-2 in RV, IAV and IBV pre-infected tissues was also qualitatively assessed by immunofluorescence assays (Figures 1G) showing less SARS-CoV-2 infected cells in co- infections vs. single infections at 3 dpi and titration (Figure S1) of SARS-CoV-2 at 3 dpi (respectively −3.31, −4.91 and −3.05 log10 in FFU/mL in RV, IAV and IBV pre- compared to non- infected tissues).

When SARS-CoV-2 was the primary infecting virus, followed by a secondary infection of RV, IAV or IBV 24 h later (Figure 1A, lower panel); RV, IAV and IBV replication was not impacted (Figures 1H and I). By infectious virus titration (Figure S1), a significant but less striking (log10 decrease in FFU 3 dpi relative to single infection −1.38) was observed only in tissues super-, compared to pre- (−4.91 log10), infected by IAV.

In summary, coinfection assays showed that SARS-CoV-2 replication is strongly or moderately impaired by a concurrent pre-existing RV/IAV infection or IBV infection, respectively, but that SARS-CoV-2 does not impair subsequent replication of RV or influenza viruses.

### Inhibition of SARS-CoV-2 correlates with induction of innate immune responses

**Figure 2.**
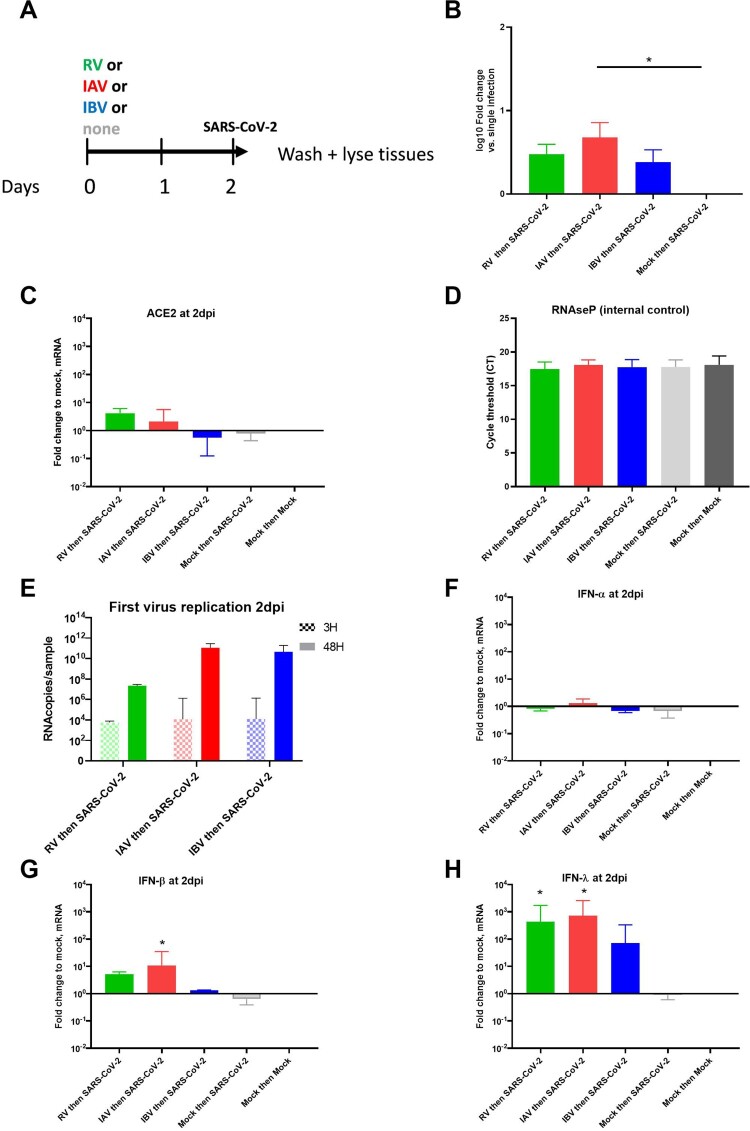
Characterization tissues at the moment of secondary infection. A. Non-infected or RV, IAV and IBV pre-infected tissues were inoculated with SARS-CoV-2 with an interval of 48h (Seasonal virus “then” SARS-CoV-2). Tissues were lysed immediately after SARS-CoV-2 inoculation and extensive washings. B. Cell-associated SARS-CoV-2 in coinfected tissues was measured by RT-PCR and represented in fold change relative to single infection. C. Expression of ACE-2 receptor in lysed tissues is represented in fold change relative to non-infected tissues and normalized to RNAseP. D. Quantification of intracellular RNAse P (housekeeping gene) from all tissues was used as an internal control. E. RV, IAV and IBV replication was quantified comparing viral load measured by RT-PCR at 3H (baseline) versus 2dpi. F, G and H. Induction of IFN-?, - ? and -?, respectively, were represented in fold change relative to non-infected tissues and normalized to RNAseP. Statistical significance was calculated using one-way ANOVA (N = 3 in all panels) (by default in comparison to non-infected tissue). *P < 0.05.

**Figure 3.**
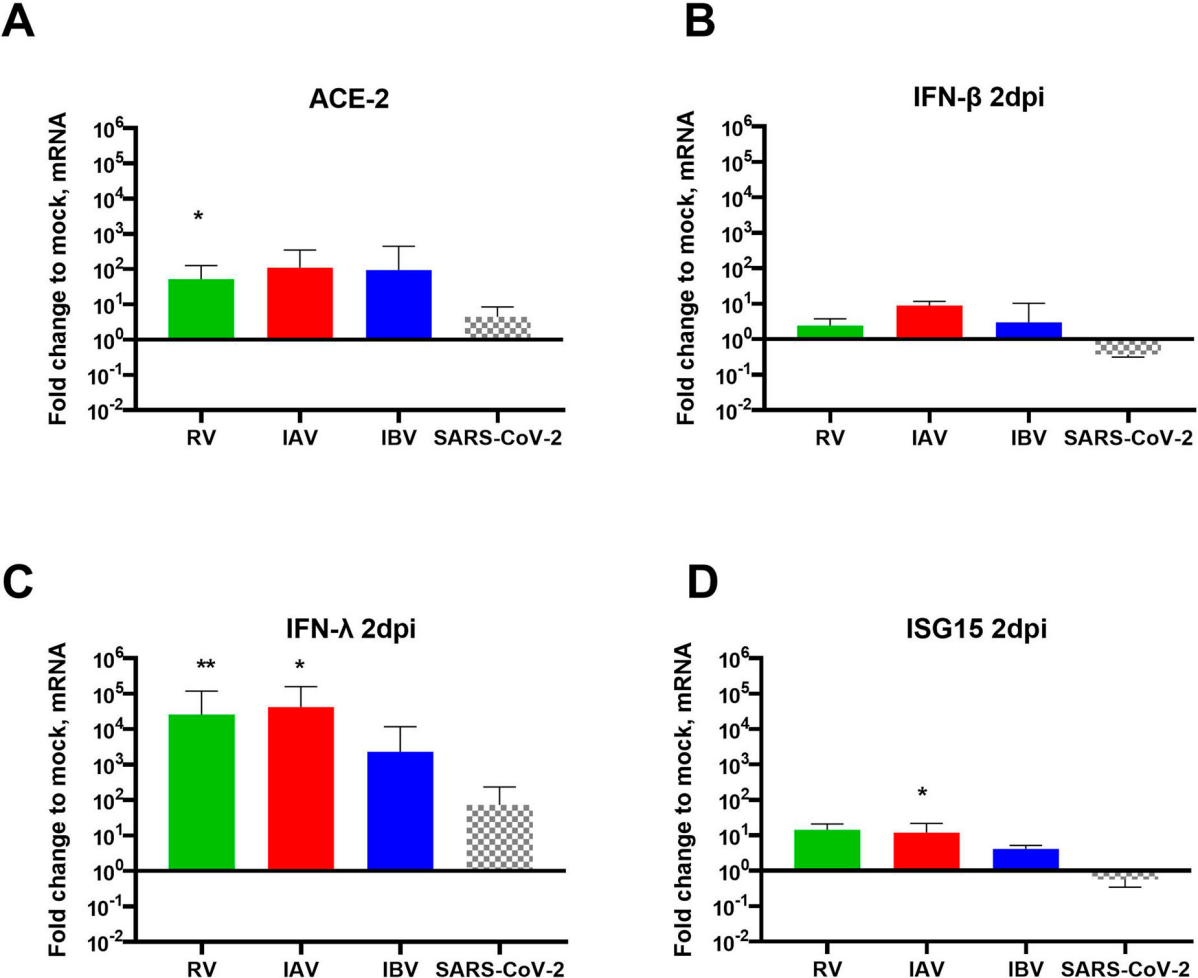
Respiratory viral infection in single and coinfections and host response at day2pi. HAE were single-infected by RV, IAV, IBV or SARS-CoV-2 and lysed at 2dpi. A to D. Induction of ACE-2 (A), IFN-? (B), -? (C) and ISG15 (D) were represented in fold change relative to non-infected and normalized to RNAseP. In comparison to induction by SARS-CoV-2, Statistical significance was calculated with t-tests (N = 3-4). In sequential infection.” *P < 0.05. **P < 0.01.

In order to understand the mechanism leading the SARS-CoV-2 reduction by a concurrent pre-existing infection with another respiratory virus, we characterized cell-bound SARS-CoV-2, ACE-2 receptor expression and epithelial innate immune responses at the moment of SARS-CoV-2 secondary infection. For this purpose, RV, IAV or IBV pre-infected tissues were inoculated with SARS-CoV-2 and immediately lysed after extensive washing (Figure 2A). Relative to single SARS-CoV-2 infections, RV, IAV or IVB pre-infected tissues had slightly higher (less than 1 log10 fold change) intracellular levels of SARS-CoV-2 RNA immediately after inoculation (Figure 2B). In these assays, to assess if higher early binding/entry of SARS-CoV-2 is associated with an upregulation of receptor expression, ACE-2 expression was measured by RT–PCR. A slight increase (log10 0.61-fold change) of ACE-2 expression compared to non-infected tissues was noted only in RV pre-infected cells (Figure 2C). Altogether, these data showed no correlation between SARS-CoV-2 entry and its inhibition by seasonal respiratory viruses. In all these experiments, the replication of the virus used for the first infection was confirmed and a RNAseP housekeeping gene was used as an internal control (Figures 2D and E). Type I (α and β) and III (λ) IFN inductions were also tested (Figures 2F, G and H). In response to RV and influenza infections, IFN-β and -λ were induced (log10 fold changes in the range of 0.12–1 for IFN-β and 1.85–2.86 log10 fold change for IFN-λ) and showed, compared to IBV, higher upregulation in RV and IAV pre-infected tissues. Of note, all virus infections induced ACE-2 at 2 dpi (mean log10 fold change relative to uninfected tissue: 1.6 for RV and IAV, 1.4 for IBV and 0.7 for SARS-CoV-2) as shown in Figure 3A. By immunostaining of tissue nuclei and ciliated cells (Figure S2), cell damage, reflected by less nuclei and ciliated cells, was only observed in tissues infected by IAV but not RV an IBV as expected [16]. To summarize this part, these data showed a better correlation of SARS-CoV-2 inhibition, by other respiratory viruses, with IFN induction rather than virus entry.

### Role of interferon induction in SARS-CoV-2 interaction with RV, IAV and IBV

In the light of these conclusions, we hypothesized that, the level of IFN response in dual infections, depending on the sequence of infections, would impact SARS-CoV-2 replication (see graphical abstract). In order to confirm this hypothesis, we therefore compared innate immunity induction by all these respiratory viruses (of this study) at 2 dpi in the context of single infections, and showed 0.5-1.4 log10 (Figures 3C), 2–3 log10 (Figures 3D), and 0.9–1.3 log10 (Figure 3E) lower induction of IFN-β, IFN-λ and IFN-stimulated gene 15 (ISG15), respectively by SARS-CoV-2 compared to common seasonal respiratory viruses. By immunofluorescence (Figure 21G) co-infected cells were barely detected in co-infected tissues, suggesting the presence of an anti-viral response that blocks the replication of SARS-CoV-2 in epithelial cells infected by the first infection.

**Figure 4.**
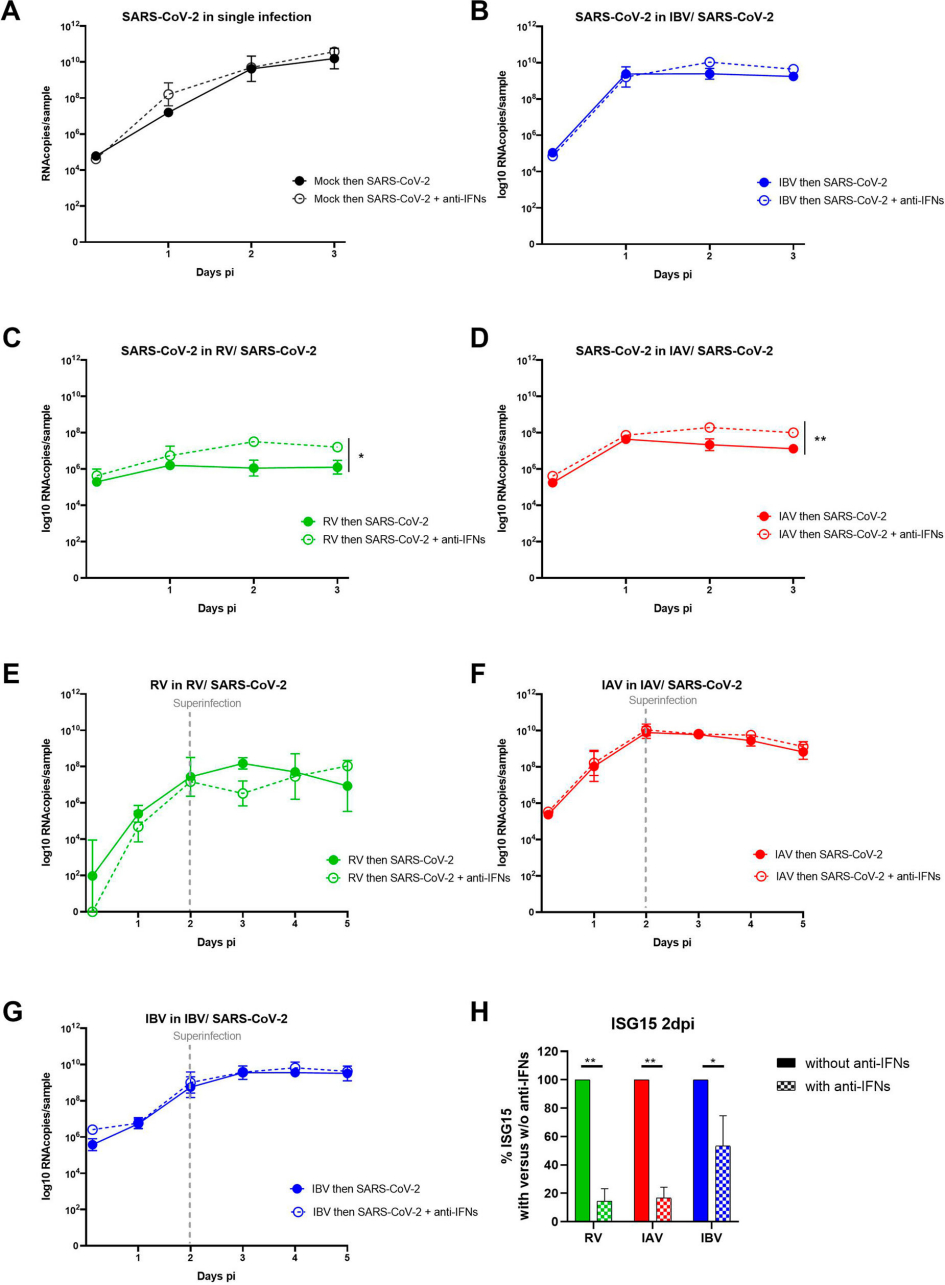
IFN involvement in SARS-CoV-2 interaction with seasonal respiratory viruses. Coinfection assays, as schemed in the upper panel of figure1A, were repeated in the presence or absence of anti-type I and III IFN neutralizing antibodies. A to G. Viral replication, similarly quantified from by RT-qPCR, was compared in the presence (dashed lines) and absence (solid lines) of anti-IFNs antibodies. A: SARS-CoV-2 in single infection. B, C and D: SARS-CoV-2 in IBV, RV and IAV-pre-infected tissues respectively. E, F and G: replication of RV, IAV and IBV in SARS-CoV-2 post-infected tissues, respectively. H. Percentage of ISG15 induction in the presence relative to in absence of anti-IFNs (dashed versus solid bars). In comparison to in the absence of anti-IFNs, statistical significance was calculated with t-tests on the area under the curve from kinetics of virus replication (A, B, C, D, E, F and G, N=3)) and using one-way ANOVA for the percentage of ISG15 induction (H, N = 2). *P < 0.05 and **P < 0.01.

To confirm the involvement of IFN in SARS-CoV-2 inhibition by a concurrent pre-existing infection with RV, IAV and IBV, similar sequential assays were then performed but in the presence of neutralizing anti-type I and III IFNs antibodies. While no effect was observed on SARS-CoV-2 in mock- and IBV pre-infected tissues (Figures 4A and B), inhibition of SARS-CoV-2 replication by a concurrent pre-existing infection with RV and IAV (Figures 4C and D) was partially but significantly rescued by IFN neutralization (1.1 and 0.9 log10 fold increase respectively). RV, IAV and IBV replications were not affected by IFN neutralization in the context of dual infections (Figures 4E, F and G). Of note, the efficiency of IFN neutralization was confirmed by the decrease of downstream ISG15 induction in these conditions (Figure 4H).

To conclude this part, these data support the involvement, at least partially, of IFN in SARS-CoV-2 inhibition by RV and IAV.

### Replication of SARS-CoV-2 Alpha variant is also inhibited by prior infection with RV or IAV and IBV

**Figure 5.**
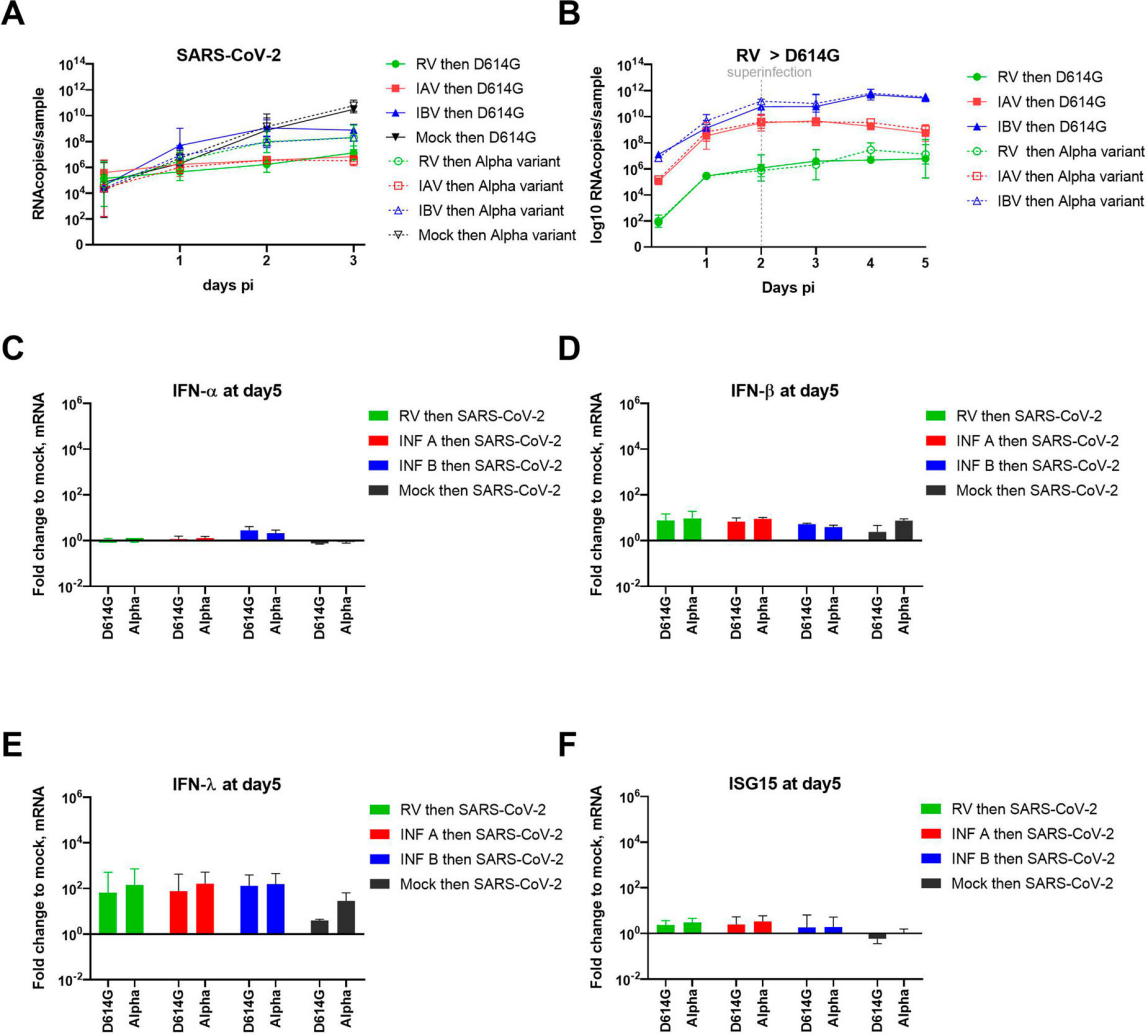
Inhibition of SARS-CoV-2 Alpha variant by prior infection with RV, IAV and IBV. Coinfection assays as schemed in the upper panel of figure1A were repeated using Alpha variant versus the first wave lineage (called “D614G”). Viral replication was similarly quantified from by RT-qPCR. A. SARS-CoV-2 replication in RV, IAV and IBV pre-infected tissues. B. RV, IAV and IBV replications in SARS-CoV-2 post-infected tissues. C, D, E and F. Induction of IFN-?, -?, -? and ISG15 respectively were represented in fold change relative to non-infected tissues and normalized to RNAseP. Data are expressed as mean and SEM (N=3 for all panels). Statistical significance was calculated with t-tests on the area under the curve from kinetics of virus replication (A and B) and using one-way ANOVA for fold change (C to F).

During the progress of this study, many variants had emerged and the Alpha VOC had outcompeted the first wave strain (B1 lineage called here “D614G”), used in all our previous single and co-infection assays. Hence, we decided to test the susceptibility of RV- IAV and IBV pre-infected tissues to this variant (Figure 5). As shown in Figure 5A, similar inhibition of the SARS-CoV-2 Alpha VOC by concurrent pre-existing infection with other respiratory viruses was observed 3 days post SARS-CoV-2 infection. Nevertheless, contrary to the observations with the first wave SARS-CoV-2, no early enhancement was observed at 1 dpi, using Alpha lineage in IBV pre-infected tissues. As D614G, superinfection with the Alpha variant had no effect on the replication of RV, IAV and IBV (Figure 5B). Despite a slight increase of IFN-β and -λ induction in SARS-CoV-2 Alpha variant induction compared to D614G lineage in single infection, IFNs and ISG15 inductions were comparable between the two variants at day 5 in the context of dual infection (Figures 5C, D, E and F).

## Discussion

As SARS-CoV-2 is entering its third winter season in the Northern Hemisphere in 2021/2022, and lifting of current public health measures is to be expected, the role of co-circulation of other respiratory viruses is of strong interest.

This work investigates SARS-CoV-2, including the Alpha VOC, and its interaction with three other seasonal respiratory viruses using the most prevalent clinical viral strains and *in vitro* differentiated airway epithelia of the upper respiratory tract. We demonstrated the effect of the presence of respiratory viruses on SARS-CoV-2 replication in the context of multiple infections. While infecting first with RV, IAV and IBV and then 48 h later with SARS-CoV-2 led to reduced SARS-CoV-2 replication even when inoculated with 2 logs higher multiplicity of infection (approximate MOI 0.1 for SARS-CoV-2 versus 0.001-0.002 for seasonal viruses), no such effect was seen when the order of infection was inverted, even when the incubation time between the two infections was shortened (in order to establish a co-infection during the exponential phase of SARS-CoV-2 infection). Our results indicate that the sequence of infection events influences the fate of SARS-CoV-2 infection. Regardless the order of infections (Figure 1A), no adaptation was observed in SARS-CoV-2 after coinfection (data not shown).

This work could be extended by assessing the interaction of SARS-CoV-2 with additional respiratory viruses like other lineages of influenza (like H3N2) and respiratory syncytial virus (RSV) and more recent SARS-CoV-2 variants. Due to technical limitations (namely the feasibility of all viral titrations), virus replication was mainly determined by the quantification of RNA viral load, which does not reflect the amount of infectious viral particles. Using FFU assays, we could globally confirm the inhibition of SARS-CoV-2 by pre-, but not a secondary, infection with seasonal respiratory viruses (Figure S1). Albeit, using this technique, a bias in SARS-CoV-2 titration because of the presence of another respiratory virus in the titrated sample is not excluded.

Findings from our investigation also unveiled mechanisms involved in SARS-CoV-2 interaction with other respiratory viruses during co-infections. We mainly provided evidence that this virus-virus interference is mediated by IFN response, which depends on the sequence of infections. In dual infections starting by a seasonal respiratory virus, SARS-CoV-2 inhibition correlated better with IFN induction by RV, IAV and IBV than virus entry rates. A limited number of co-infected cells was observed in co-infected tissues. Anti-IFN neutralizing anti-bodies could partially prevent SARS-CoV-2 inhibition during these sequential infections. SARS-CoV-2, sensitive to IFN response [23,24], appears inhibited by the anti-viral state induced by RV and influenza viruses. In contrast, in inverted infection sequence, SARS-CoV-2 was able to replicate before there was any interference due to IFN induction by a second infection with RV, IAV or IBV. Furthermore, thanks to its IFN antagonistic effect (reviewed in [25]), the low IFN induction by SARS-CoV-2, as previously demonstrated [24] and confirmed here in Figures 3B, C and D, would explain why this coronavirus had no effect on the replication of the three other respiratory viruses. The capacity to antagonize IFN response has been already described for a number of coronaviruses and has been considered as a virulence factor (reviewed in [26]).

In order to overcome any bias and variability due to the genetic and environmental background of individual donors, we here used a standardized model (as mentioned in the method section).

Our data were reproducible in several independent experiments (N = 3–6), which supports the robustness of our conclusions and also corroborate recent paper showing that RV replication abolished SARS-CoV-2 infection though IFN pathway [21]. In contrast to this study, Dee *et al* did not find that infection sequence mattered. These differing results could be explained by different conditions of coinfection assay, such as the MOI (0.05 for both viruses, which was higher for RV and lower for SARS-CoV-2 compared to our experiments) and the incubation temperature (at 37°C instead of 33°C), which can affect IFN response as previously described [27]. Here we performed all infections at 33°C, as the optimal growth temperature for RV and SARS-CoV-2 [28,29]. IFN induction constitutes the first efficient non-specific immune response against respiratory viral infection in airway epithelia. Its involvement in virus-virus interactions has been recently described for a number of respiratory viruses [17,18,30]. In patients, differing levels of COVID-19 severity have been associated with impaired IFN and inflammatory responses [31]. Nevertheless, contrary to what has been suggested earlier [32], IFN treatment is unlikely to be considered for therapeutic purposes against COVID-19. First, in SARS-CoV-2 infection, interferon induction seems also to play a key role in driving the pathology [33]. Second, a recent *in vitro* study of the resistance of SARS-CoV-2 variants to IFN pre-treatment suggested that the escape from the innate immune response would be a driving force of SARS-CoV-2 evolution leading to the emergence of lineages with increasing resistance to antiviral IFN response [34].

The partial rescue of SARS-CoV-2 replication in RV and IAV pre-infected tissues when using neutralizing anti-IFN antibodies could be explained by the incomplete inhibition of IFN induction, as shown in Figure 4H. It would also support the involvement of additional host pathways like endocytosis, multivesicular bodies and autophagy [35–37] in virus-virus interactions. Further investigation is still needed to confirm and assess their implications. In the presence of IAV, the loss of epithelial cells ([16] and Figure S2) could also contribute to the inhibition of SARS-CoV-2. The involvement of more than one mechanism in IBV-SARS-CoV-2 interaction has been also suggested by the increase of IBV pre-infected tissues permissiveness to D614G, but not the Alpha variant, SARS-CoV-2 24 h after its inoculation (Figures 1B and 4A). This enhancement likely implicates the early steps of SARS-CoV-2 infection rather than IFN induction (Figure 5C to F). It would also depend on the virus inoculated during the first infection and SARS-CoV-2 lineage, as observed in some IBV/SARS-CoV-2 D614G co-infections. Dissecting the mechanism involved necessitates deeper investigations. It has been recently shown that experimental infection of cells with IAV amplified the expression of molecules necessary for SARS-CoV-2 infection of the distal lung such as the SARS-CoV-2 receptor ACE-2 and the host protease TMPRSS [38]. Bai L. *et al* recently showed the IAV promotion of SARS-CoV-2 replication in co-infected standard cells and mice was associated with upregulation and ACE-2 and was independent of the IFN response [39]. In our study using a more relevant model to mimic infection in patient airway epithelia, we confirmed the increase of ACE-2 expression in HAE by IAV, but without any correlation with virus-virus interaction.

Even though a preceding infection with a seasonal respiratory virus might attenuate SARS-CoV-2 infection and could thus theoretically lead to lower infection rates or mitigate clinical disease, infection prevention measures that are effective against all respiratory viruses are of utmost importance as long as high circulation of SARS-CoV-2 is seen. During the pandemic, vaccination against influenza viruses was highly recommended to avoid more overload of hospitals with SARS-CoV-2 and influenza patients. In the prospect of co-circulation of influenza viruses and SARS-CoV-2, our results are encouraging that co-infection would not lead to more pronounced replication of either virus. Although the sequence of infection events will be difficult to determine in real-life, more data on clinical course and outcomes of co-infected individuals are needed to better understand long-term consequences of SARS-CoV-2 and other respiratory viruses’ co-circulation at the population level. This will also depend on the circulating strains during the next season, the seasonality of respiratory viruses and the patients’ immune background against these viruses. Furthermore, in this work we studied coinfections focusing on the involvement of host response in virus-virus interaction. Other aspects would be also be crucial to shape SARS-CoV-2 epidemiology, such as its transmissibility in co-infected patients that might be facilitated by the presence of respiratory symptoms like coughing and sneezing or contained by the non-pharmaceutical interventions. The loss of ciliated cells, frequently observed in patients infected by influenza virus, could also prevent additional infection by SARS-CoV-2. It is also noteworthy to mention that our model only recapitulates the early steps of an acute infection before the trigger of the adaptive immunity and the without the participation of the immune cells. However, it also mimics persistent infections in immune-compromised patients [16].

In conclusion, this deep investigation of SARS-CoV-2 interaction with seasonal respiratory viruses in an *ex-vivo* model extends our knowledge about co-infections of the upper respiratory tract. Beyond the highlight of the host pathways implicated during airway epithelium coinfection, this study might help improve our understanding and prediction of SARS-CoV-2 replication in co-infected patients.

## Supplementary Material

Supplemental Material
