# Supplementary material for: Sequential infections with rhinovirus and influenza modulate the replicative capacity of SARS-CoV-2 in the upper respiratory tract
Source: Emerg Microbes Infect. 2022 Jan 27;11(1):412–23. doi: 10.1080/22221751.2021.2021806 (PMC8803056; doi:10.1080/22221751.2021.2021806)
Supplement: Supplemental Material [file TEMI_A_2021806_SM9060.docx]

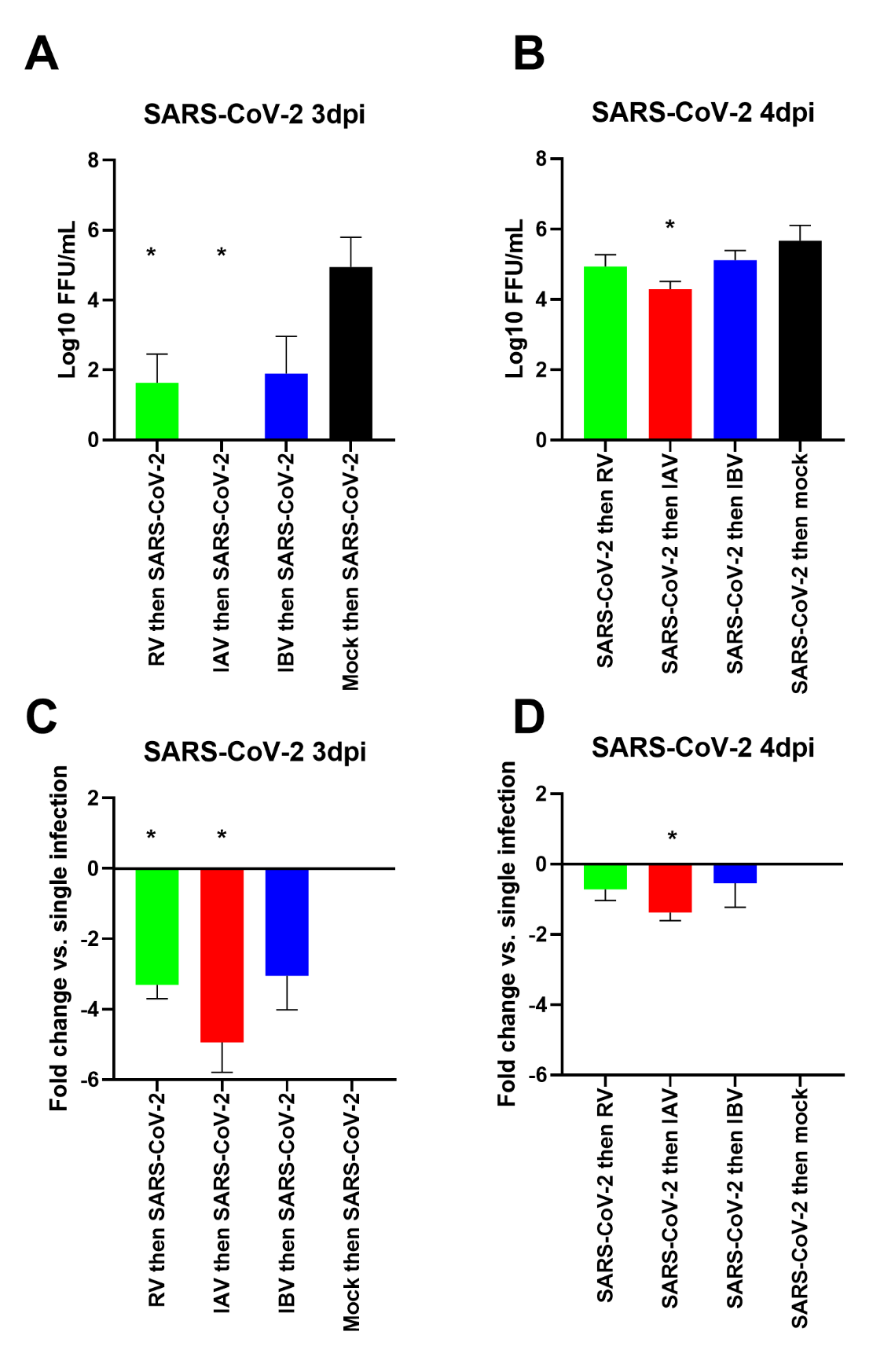


**Figure S1: SARS-CoV-2 infectious titer in single versus dual infections in HAE.** From the same dual and single infection assays performed in figure1 in HAE, infectious titer of apically released SARS-CoV-2 was assessed (see material and method) and represented in FFU/mL (FFU: focus-forming Unit) (A and B respectively) and Fold change relative to single infection (C and D respectively). A and C show SARS-CoV-2 replication at day 5 in tissues pre-infected by seasonal viruses (as schemed in the upper panel of the figure 1A). B and D shows SARS-CoV-2 replication at day 4 in tissues with a secondary infection by RV, IAV and IBV (as schemed in the lower panel of the figure 1A). In comparison to single infection, statistical significance was calculated using t-tests (N=3). *P < 0.05.

**
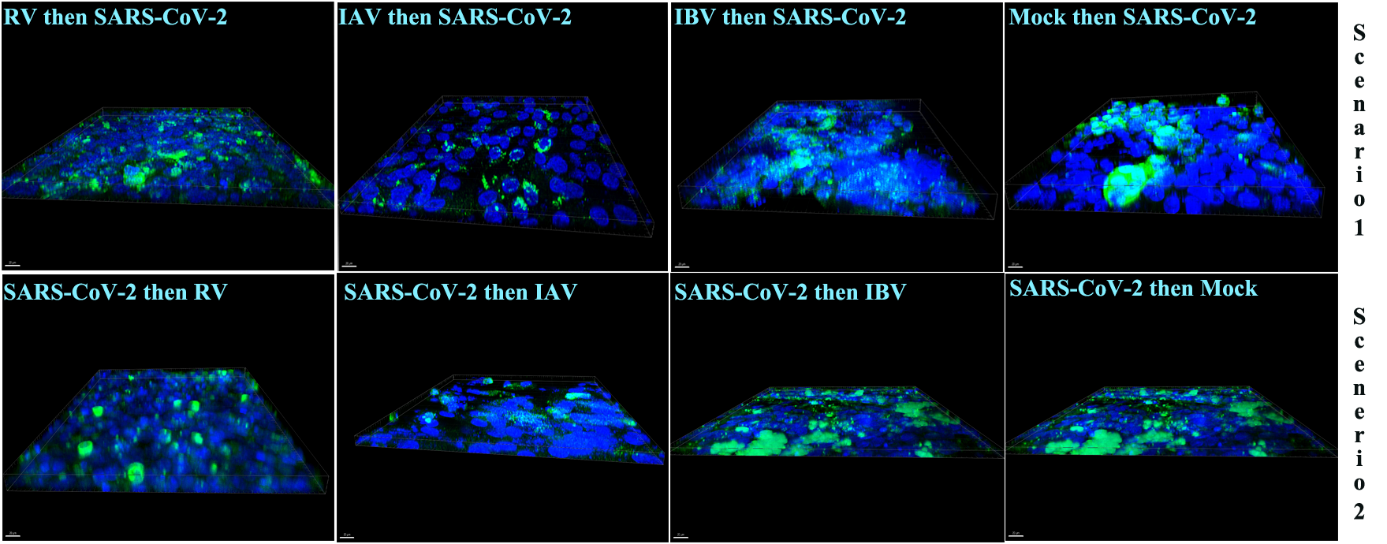
Figure S2: Tissue integrity in single versus dual infections.** At day 5 (in case of the 1st scenario, upper panel) and day 4 (in case of the 2nd scenario, lower panel), tissues from single and dual infection assays as in figure 1 were immuno-stained using antibodies against β-tubulin IV and DAPI as a marker of ciliated cells (green) and nuclei (Blue) respectively. Images acquired by confocal microscopy (4 sections/ tissue) were processed by Imaris software for 3D reconstruction.

| **Virus** | | **Sequence** | **Study** | **MOI** |
| --- | --- | --- | --- | --- |
| SARS-CoV-2 | D614G (B1) | hCoV-19/Switzerland/GE-SNRCI-29943121/2020 (in GISAID) | (40) | 0.1 |
| SARS-CoV-2 | Alpha (B1.1.7) | hCoV-19/Switzerland/2012212272/2020 (in GISAID) | (41) | 0.1 |
| RV | RV-A16 | Not available | (17) | 0.002 |
| IAV | H1N1 pan2009 | Not available | This study | 0.001 |
| IBV | B/Switzerland/6330/2020 (Victoria) | Not available | This study | 0.001 |

**Table S1: information about viruses used in this study.** Viral stocks were isolated and produced directly from clinical specimens in HAE. For each virus, the multiplicity of infection (MOI) was selected in the range where the virus has the optimal kinetics in single infection. MOI are approximately calculated considering that the number of accessible cells in HAE is around 200000 (Essaidi-Laziosi M et al 2017). SARS-CoV-2 titration was performed in Vero E6 cells (monkey kidney), RV in Mucilair^TM^ tissue and influenza viruses in MDCK cells (Madin-Darby Canine Kidney).

**Supplementary references**

40. Vetter P, Eberhardt CS, Meyer B, Martinez Murillo PA, Torriani G, Pigny F, et al. Daily viral kinetics and innate and adaptive immune response assessment in COVID-19: A case series. Msphere. 2020;5(6):e00827-20.

41. Bekliz M, Adea K, Essaidi-Laziosi M, Sacks JA, Escadafal C, Kaiser L, et al. SARS-CoV-2 rapid diagnostic tests for emerging variants. The Lancet Microbe. 2021.
